# Androgen regulation of gene expression in human meibomian gland and conjunctival epithelial cells

**Published:** 2012-04-27

**Authors:** Payal Khandelwal, Shaohui Liu, David A. Sullivan

**Affiliations:** Schepens Eye Research Institute and Department of Ophthalmology, Harvard Medical School, Boston, MA

## Abstract

**Purpose:**

Androgens exert a significant influence on the structure, function and/or pathophysiology of the meibomian gland and conjunctiva. We sought to determine whether this hormone action involves the regulation of epithelial cell gene expression in these tissues.

**Methods:**

Immortalized human meibomian gland and conjunctival epithelial cells were treated with placebo or dihydrotestosterone (DHT) and processed for molecular biologic procedures. Gene expression was evaluated with BeadChips and data were analyzed with bioinformatic and statistical software.

**Results:**

Androgen treatment significantly influenced the expression of approximately 3,000 genes in immortalized human meibomian gland and conjunctival epithelial cells. The nature of DHT action on gene activity was predominantly cell-specific. Similarly, DHT exerted a significant, but primarily cell-specific, influence on many gene ontologies and Kyoto Encyclopedia of Genes and Genomes (KEGG) pathways. These included groups of genes related, for example, to lipid dynamics, innate immunity, cell cycle, Janus kinase (JAK)-signal transducer and activator of transcription (stat) cascades, oxidative phosphorylation, the proteasome, and mammalian target of rapamycin (mTOR), Wnt, and peroxisome proliferator-activated receptor (PPAR) signaling.

**Conclusions:**

Our findings support our hypothesis that androgens regulate gene expression in human meibomian gland and conjunctival epithelial cells. Our ongoing studies are designed to determine whether many of these genes are translated and play a role in the health and well being of the eye.

## Introduction

Androgens exert a significant influence on the structure, function and/or pathophysiology of many ocular tissues, including the meibomian gland, lacrimal gland, conjunctiva, and cornea [1-12]. These hormones regulate such ocular parameters as glandular architecture, protein synthesis and secretion, meibum production, mucus expression, aqueous tear output, tear film stability, immune activity, and epithelial cell dynamics [1-12]. Androgens have also been reported to correct defects, facilitate wound healing [6,7,13], suppress angiogenesis [14], and stimulate mitosis [9] in the corneal epithelium, to alter the development of allergic conjunctivitis [5], and to attenuate inflammation in autoimmune lacrimal tissue [8,11]. In addition, androgens have been proposed as a topical therapy for the treatment of aqueous-deficient and evaporative dry eye diseases [8,11]. However, despite these observations, the precise mechanisms underlying androgen-eye interactions in humans remain to be clarified.

We hypothesize that androgen action on the eye involves the local, intracrine synthesis of this sex steroid from adrenal precursors (e.g., dehydroepiandrosterone), binding to saturable, high-affinity and androgen-specific receptors, control of gene transcription, and ultimately modulation of translation. In support of this hypothesis, we have discovered that the human meibomian and lacrimal glands, and immortalized corneal and conjunctival epithelial cells, contain all the steroidogenic enzyme mRNAs necessary for the intracrine synthesis and metabolism of androgens [15]. Moreover, we have shown that androgen receptor mRNA and protein are present in epithelial cell nuclei of the human meibomian and lacrimal glands, cornea and conjunctiva [16,17].

To continue to test our hypothesis, we examined the influence of androgens in gene expression in immortalized human meibomian gland and conjunctival epithelial cells.

## Methods

### Cell culture and hormone treatment

Immortalized human meibomian gland epithelial cells, which were recently generated in our laboratory [2], were cultured in Keratinocyte Serum-Free Medium [KSFM] supplemented with 50 μg/ml bovine pituitary extract (BPE), 5 ng/ml epidermal growth factor (EGF), and 100 U penicillin-streptomycin (Invitrogen, Carlsbad, CA). Cells were incubated in a humidified, 37 °C chamber under 5% CO_2_/95% air. Immortalized human conjunctival epithelial cells, which were gifted by Dr. Ilene Gipson (Boston, MA), were cultured in serum-free conditions as previously described [18].

When approximately 80% confluent, cells were exposed to 10 nM dihydrotestosterone (DHT; Steraloids, Wilton, NH) or placebo for 3 (meibomian) or 4 (conjunctiva) days. These time periods were previously shown to be optimal for the generation of DHT-induced alterations in androgen receptor mRNA levels in the different cell types [19]. For these studies the DHT was dissolved in ethanol and aliquots were evaporated in sterilized vials before the addition of medium. The placebo was prepared by transferring media to vials containing the residue of evaporated ethanol. After hormone treatment, cells were harvested and processed for RNA isolation.

### Molecular biologic procedures

Total RNA was extracted with RNAqueous Kits (Ambion, Austin, TX) and evaluated on a RNA Nano 6000 Series II Chip with a 2100 Bioanalyzer (Agilent Technologies, Palo Alto, CA) to confirm RNA integrity. The RNA concentrations and associated 260/280 nm ratios were determined using a NanoDrop 1000 Spectrophotometer (Thermo Scientific, Waltham MA).

The RNA (100 ng) samples were processed by Asuragen (Austin, TX) for the determination of mRNA levels by using Illumina HumanHT-12 v3 Expression BeadChips (San Diego, CA).These BeadChips target more than 25,000 annotated genes with over 48,000 probes derived from NCBI reference sequences and the UniGene databases. In brief, biotin-labeled cRNA samples were generated by using a MessageAmp™ II-based protocol (Ambion Inc., Austin, TX), quantitated by UV spectrophotometry and analyzed with an Agilent 2100 Bioanalyzer capillary electrophoresis system. The labeled cRNAs were used to probe the BeadChips. Hybridization, washing, and scanning of the Illumina arrays were conducted according to the manufacturer’s instructions. Data were processed with Illumina BeadStudio software v3 by using both background subtraction and cubic spline normalization. Standardized hybridization intensity values were adjusted by adding a constant, so that the lowest intensity value for any sample equaled 16 [20].

Normalized data were analyzed with GeneSifter.Net software (Geospiza, Seattle, WA), a comprehensive program that also produced gene ontology and z-score reports. Ontologies included biologic processes, molecular functions and cellular components and were organized according to the guidelines of the Gene Ontology Consortium (GO) [21]. Gene expression data were analyzed with and without log transformation and statistical analyses of these data were performed with Student’s *t*-test (two-tailed, unpaired). Genes that were up- or down-regulated in the same direction in different experiments were identified by using the GeneSifter.Net intersector program (Geospiza). All data from the Illumina BeadChips are accessible for download through the National Center for Biotechnology Information’s Gene Expression Omnibus (GEO) via series accession numbers (GSE18091 and GSE18094).

### Real time PCR procedures

The differential expression of selected genes was verified by using quantitative real-time PCR (qPCR) procedures. The cDNAs were transcribed by employing SuperScript III Reverse Transcriptase (Invitrogen, Grand Island, NY) and random hexamer primers (Invitrogen). The qPCR reactions were performed in triplicate by using TaqMan Gene Assays (Applied Biosystems, Inc., Foster City, CA) and TaqMan-specific primers and probes for aldo-keto reductase family 1, member c2 (Hs00413886_m1*), cdc28 protein kinase regulatory subunit 2 (Hs01048812_g1*), EGF-containing fibulin-like extracellular matrix protein 1 (Hs002444575_m1*), interferon α-inducible proteins 6 (Hs00242571_m1*), kallikrein related peptidase 11 (Hs01100849_m1*), keratin 16 (Hs00373910_g1*),

laminin, α3 (Hs00165042_m1*), leupaxin (Hs00183105_m1*), minichromosome maintenance component 3 (Hs00172459_m1*), myosin light chain 6 (Hs00819642_m1; conjunctival epithelial cell endogenous control), n (α) acetyltransferase 50 (Hs00363889_m1*; meibomian gland epithelial cell endogenous control), plasminogen activator, urokinase (Hs00170182_m1), serum amyloid A1 (Hs00761940_s1), and uridine phosphorylase 1 (Hs00427695_m1*). Differential gene expression was calculated according to the Comparative Ct method, as outlined in Applied Biosystems User Bulletin 2 (updated 2001).

## Results

### Androgen impact on gene expression in human ocular surface and meibomian gland epithelial cells

To determine the effect of DHT on gene expression in immortalized human meibomian gland and conjunctival epithelial cells, cells were exposed to placebo or DHT and processed for analysis by using Illumina BeadChips and Geospiza software.

Our results demonstrate that DHT had a significant impact on the expression of approximately 3,000 genes in immortalized human meibomian gland and conjunctival epithelial cells (Table 1). The relative direction of this hormone effect was about the same in both cell types, with DHT up- and down-regulating similar percentages of genes (i.e., meibomian: 49.8% ↑; conjunctiva: 44.8% ↑). Examples of genes that showed notable hormone-induced differences in terms of ratios are listed in Table 2 and Table 3. In addition, DHT significantly enhanced the expression of genes encoding mucin 16 (2.2 fold ↑, conjunctiva) and reduced the activity of genes for S100 calcium binding proteins A8 and A9 (2.1 and 1.4 fold ↓, respectively, conjunctiva). Analysis of BeadChip raw data also revealed that DHT caused an 8.0 and 39.7 fold decrease in the mRNA levels of the small proline-rich proteins 2F and 2A, respectively, in meibomian gland epithelial cells.

**Table 1:** Influence of DHT on gene expression in human meibomian gland and conjunctival epithelial cells.

**Immortalized human epithelial cell type**	**DHT>Placebo**	**Placebo>DHT**	**Total**
Meibomian gland	1,485	1,494	2,979
Conjunctiva	1,350	1,662	3,012

Gene expression was significantly (p<0.05) upregulated in cells exposed to DHT or placebo treatment, as shown by the analysis of log-transformed data.

**Table 2:** Effect of DHT on gene expression ratios in immortalized human meibomian gland epithelial cells.

**Accession #**	**Gene**	**Ratio**	**p value**	**Ontology**
**DHT>Placebo**				
NM_001001552	LEM domain containing 1	10.1	0.00000	nuclear envelope
NM_032563	Late cornified envelope 3D	4.5	0.00024	keratinization
NM_152565	ATPase, H^+^ transporting, lysosomal 38 kDa, V0 subunit d2	3.7	0.00048	ion transport
NM_021244	Ras-related GTP binding D	3.5	0.00010	positive regulation of TOR signaling cascade
NM_001031615	Aldehyde dehydrogenase 3 family, member B2	3.0	0.00004	alcohol metabolic process
NM_000435	Notch homolog 3	2.9	0.00015	regulation of transcription, DNA-dependent
NM_005218	Defensin, β1	2.6	0.00001	chemotaxis
NM_001003679	Leptin receptor	2.5	0.00553	energy reserve metabolic process
NM_001047	Steroid-5α-reductase, α polypeptide 1	1.9	0.00097	androgen biosynthetic process
NM_005063	Stearoyl-CoA desaturase	1.7	0.00039	fatty acid biosynthetic process
NM_002015	Forkhead box O1	1.6	0.00006	blood vessel development
**Placebo>DHT**				
NM_181800	Ubiquitin-conjugating enzyme E2C	24.4	0.00000	cell cycle checkpoint
NM_001067	Topoisomerase (DNA) II α 170 kDa	16.7	0.00000	resolution of meiotic recombination intermediates
NM_018454	Nucleolar and spindle associated protein 1	15.6	0.00006	mitotic sister chromatid segregation
NM_001255	Cell division cycle 20 homolog	13.4	0.00001	cell cycle checkpoint
NM_001786	Cyclin-dependent kinase 1	12.3	0.00000	cell cycle checkpoint
NM_001168	Baculoviral IAP repeat-containing 5	10.2	0.00000	G2/M transition of mitotic cell cycle
NM_004701	Cyclin B2	9.5	0.00002	cell cycle checkpoint
NM_002263	Kinesin family member C1	9.0	0.00000	mitotic sister chromatid segregation
NM_002994	Chemokine (C-X-C motif) ligand 5	8.9	0.00019	chemotaxis
NM_003246	Thrombospondin 1	4.7	0.00001	activation of MAPK activity
NM_002727	Serglycin	2.5	0.00012	apoptosis
NM_004994	Matrix metallopeptidase 9	1.7	0.00001	proteolysis

Relative ratios were calculated by comparing the degree of gene expression in meibomian gland epithelial cells treated with placebo or DHT. The mean gene intensity level in at least one group exceeded 100 BeadChip units.

**Table 3:** Influence of DHT on gene expression ratios in immortalized human conjunctival epithelial cells.

**Accession #**	**Gene**	**Ratio**	**p value**	**Ontology**
**DHT>Placebo**				
NM_004994	Matrix metallopeptidase 9	10.9	0.00000	proteolysis
NM_001012964	Kallikrein-related peptidase 6	10.5	0.00001	proteolysis
NM_001323	Cystatin E/M	10.4	0.00002	epidermis development
NM_003856	Interleukin 1 receptor-like 1	9.0	0.00006	immune response
NM_018043	Anoctamin 1, calcium activated chloride channel	8.0	0.00001	ion transport
NM_002153	Hydroxysteroid (17β) dehydrogenase 2	7.8	0.00000	steroid biosynthetic process
NM_005416	Small proline-rich protein 3	7.4	0.00023	epidermis development
NM_144947	Kallikrein-related peptidase 11	7.2	0.00003	proteolysis
NM_001077491	Kallikrein-related peptidase 5	7.1	0.00018	proteolysis
NM_198129	Laminin, α3	5.6	0.00001	epidermis development
**Placebo > DHT**				
NM_002993	Chemokine (C-X-C motif) ligand 6	12.4	0.00005	chemotaxis
NM_003186	Transgelin	10.8	0.00013	muscle organ development
NM_002974	Serpin peptidase inhibitor, clade B, member 4	10.1	0.00000	immune response
NM_001733	Complement component 1, r subcomponent	9.4	0.00012	proteolysis
NM_005602	Claudin 11	8.8	0.00005	cell adhesion
NM_006820	Interferon-induced protein 44-like	8.5	0.00001	immune response
NM_016352	Carboxypeptidase A4	8.4	0.00033	proteolysis
NM_003641	Interferon induced transmembrane protein 1	8.0	0.00001	cell surface receptor linked signaling pathway
NM_001710	Complement factor B	7.7	0.00000	proteolysis
NM_001044391	Mucin 1, cell surface associated	3.1	0.00127	protein binding

Relative ratios were determined by comparing the degree of gene expression in conjunctival epithelial cells treated with placebo or DHT. The mean gene intensity level in at least one group was higher than 100 BeadChip units.

Genes that demonstrated the greatest alterations in terms of statistical significance included those increased or decreased by DHT in immortalized human meibomian gland (aldo-keto reductase family 1, member C2 ↑, p<0.000001; DNA topoisomerase IIα ↓, p<0.000001), and conjunctival (uridine phosphorylase 1 ↑, p<0.000001; interferon, α-inducible protein 6 ↓, p<0.000001) epithelial cells.

The nature of androgen action on gene expression was predominantly cell-specific. Thus, 61.0 and 53.6% of upregulated genes, and 58.1 and 52.0% of downregulated genes, were unique to the meibomian gland and conjunctival epithelial cells, respectively. In addition, between 12.9 to 20.0% of regulated genes were expressed in the opposite direction in these immortalized cells (Table 4).

**Table 4:** Opposite effects of DHT on gene expression in immortalized human meibomian gland and conjunctival epithelial cells.

**Cell 1**	**Cell 2**	**C1 ↑, C2 ↓ (Genes)**	**%**	**C1 ↓, C2 ↑ (Genes)**	**%**
Meibomian	Conjunctiva	199	12.9–14.2	255	18.3–20.0

Log transformed data were analyzed and the total number of genes with GEO sequence identities in each category was then determined. Gene expression was significantly (p<0.05) up (↑)- or down (↓)-regulated by DHT in the specific cell type. Abbreviations: “C” stands for “cell.”

The genes regulated by DHT were located on a variety of chromosomes. As shown in Table 5, the cellular pattern of this regulation showed some similarities and dissimilarities.

**Table 5:** Effect of DHT on chromosomal gene expression in immortalized human meibomian gland and conjunctival epithelial cells.

**Chromosome**	**DHT Genes ↑**	**Plac Genes ↑**	**DHT z-score**	**Plac z-score**
**Meibomian gland**				
16	66	53	**3.35**	1.34
19	80	62	**2.61**	0.2
18	34	17	**2.13**	−1.44
2	73	95	**-2.23**	0.21
17	69	77	1.52	**2.65**
22	25	35	0.42	**2.59**
8	51	32	−0.08	**-2.83**
13	23	14	−1.23	**-2.93**
**Conjunctiva**				
16	66	45	**4.08**	−0.49
20	51	37	**3.84**	0.18
1	143	147	**3.51**	1.77
19	73	73	**2.42**	0.94
8	32	40	**-2.33**	**-2.22**
13	15	19	**-2.39**	**-2.37**
3	47	94	**-2.57**	1.77
X	24	46	**-2.63**	−0.19
4	35	60	**-2.72**	−0.51
5	34	82	**-3.6**	1.27
12	46	92	−1.53	**3.15**
17	51	85	−0.31	**2.94**
15	34	26	0.2	**-2.13**
7	64	44	1.51	**-2.51**
2	88	75	0.27	**-2.76**

Chromosomes with the highest and lowest z-scores were selected after analysis of log-transformed Illumina BeadChip data. A z-score is a statistical rating of the relative expression of genes, and shows how much they are over- or under-represented in a given gene list [59]. Positive z scores represent a greater number of genes meeting the criterion than is expected by chance, whereas negative z scores reflect fewer genes meeting the criterion than expected by chance [59]. Z-scores with values >2.0 or <-2.0 are quite significant and are highlighted in bold print. Terms: DHT Genes ↑ - number of genes upregulated in DHT-treated cells; Plac Genes ↑ - number of genes upregulated in placebo-treated cells; z-score - specific score for the upregulated genes in the DHT- and placebo-exposed cells.

To confirm in part the Illumina BeadChip results, selected genes were analyzed by qPCR. This experimental approach verified the alterations of all tested genes (Table 6).

**Table 6:** Confirmation of selected Illumina BeadChip chip results by qPCR.

**Gene**	**Illumina ratio**	**qPCR ratio**
**Meibomian Gland Epithelial Cells**		
**DHT>Placebo**		
Keratin 16	2.4	5.7
Aldo-keto reductase family 1, member C2	2.6	1.7
Kallikrein-related peptidase 11	1.8	2.4
**Placebo>DHT**		
CDC28 protein kinase regulatory subunit 2	3.1	1.5
Minichromosome maintenance component 3	3.5	19.3
Leupaxin	3.1	57.8
**Conjunctival Epithelial Cells**		
**DHT>Placebo**		
Laminin, α3	5.6	6.9
Plasminogen activator, urokinase	5.3	1.1
Uridine phosphorylase 1	4.2	2.7
**Placebo>DHT**		
Interferon, α-inducible protein 6	4.8	1.6
Serum amyloid A1	5.7	3.1
EGF-containing fibulin-like extracellular matrix protein 1	4.9	2.5

The expression of designated genes, that were shown to be significantly altered in DHT-treated cells by using Illumina BeadChips, were re-examined with qPCR procedures. The qPCR data from meibomian gland cells were standardized to N (α) acetyltransferase B complex 50 and data from conjunctival cells were normalized to myosin, light chain 6, alkali, smooth muscle and non-muscle. Neither of the genes used for standardization responded to DHT exposure. The relative ratios of gene expression in 3 separate experiments are listed in the Illumina and qPCR “Ratio” columns.

### Androgen influence on the expression of gene ontologies and Kyoto Encyclopedia of Genes and Genomes (KEGG) pathways in human ocular surface and meibomian gland epithelial cells

Androgen administration had a significant impact on the biologic process, molecular function and cellular component gene ontologies, as well as on the KEGG pathways, in human ocular surface and meibomian gland epithelial cells.

As shown in Table 7, DHT significantly increased numerous ontologies in immortalized human meibomian gland epithelial cells, such as those associated with protein metabolism, signaling, tissue development, oxidoreductase and peptidase activities, intracellular organelles and peroxisomes. Treatment with DHT also stimulated 25 different ontologies (with ≥5 genes) concerned with lipid biosynthesis, homeostasis, transport and binding, as well as with cholesterol, fatty acid, phospholipid and steroid dynamics, as we recently reported [2]. In turn, DHT decreased ontologies linked to cell cycle, M phase, DNA metabolic processes, angiogenesis, innate immunity, RNA binding, and ribonucleoprotein complexes. These effects of DHT were paralleled by significant alterations in KEGG pathways. Androgen exposure upregulated pathways related to insulin, mTOR and peroxisome proliferator-activated receptor (PPAR) signaling, and downregulated those involved with the cell cycle, RNA transport and cancer (Table 8).

**Table 7:** Influence of DHT on the expression of gene ontologies in human meibomian gland epithelial cells.

**Ontology**	**DHT Genes ↑**	**Plac Genes ↑**	**DHT z-score**	**Plac z-score**
**Biologic Process**				
programmed cell death	136	143	**5.18**	**5.09**
oxidation-reduction process	95	61	**4.85**	−0.42
protein metabolic process	280	278	**4.35**	**2.85**
purine ribonucleotide metabolic process	50	37	**3.8**	0.87
nerve growth factor receptor signaling pathway	28	22	**3.38**	1.44
response to hormone stimulus	61	48	**3.36**	0.7
regulation of signaling	132	106	**3.14**	−0.45
tissue development	80	69	**2.58**	0.46
cell cycle	75	202	−0.65	**13.58**
M phase	14	107	**-3.29**	**13.34**
DNA metabolic process	38	118	−0.87	**11.17**
organelle fission	12	81	**-2.41**	**11.94**
RNA processing	39	111	−0.49	**10.47**
angiogenesis	21	30	1.05	**2.95**
immune system process	99	122	0.86	**2.66**
innate immune response	27	41	−0.08	**2.28**
blood vessel development	30	35	1.49	**2.15**
regulation of cellular biosynthetic process	139	148	**-2.24**	**-2.39**
regulation of transcription	111	119	**-2.26**	**-2.33**
neurologic system process	59	33	**-3.68**	**-7.15**
**Molecular Function**				
protein binding	572	707	**6.41**	**13.12**
hydrogen ion transmembrane transporter activity	21	3	**5.82**	−1.58
oxidoreductase activity, acting on CH-OH group of donors	23	9	**5.47**	0.17
catalytic activity	423	403	**5.11**	**2.27**
translation factor activity, nucleic acid binding	16	8	**4.47**	0.82
peptidase activity	55	36	**3.01**	−0.63
RNA binding	61	127	1.12	**10.03**
nucleotide binding	164	243	1.32	**7.58**
ATPase activity	31	42	1.61	**3.59**
helicase activity	7	20	−0.89	**3.2**
ion channel activity	14	12	**-2.51**	**-3.15**
DNA binding	115	149	**-2.82**	−0.43
zinc ion binding	121	99	−0.68	**-3.47**
transporter activity	82	50	0.41	**-3.94**
transmembrane receptor activity	33	26	**-5.76**	**-6.98**
**Cellular Component**				
vacuole	56	20	**7.82**	−0.56
lysosome	45	20	**6.54**	0.19
intracellular organelle	721	852	**5.76**	11.82
organelle membrane	191	152	**5.26**	0.86
endoplasmic reticulum	109	76	**4.97**	0.28
peroxisome	19	4	**4.85**	−1.23
nuclear part	165	335	−0.05	**13.77**
organelle lumen	195	344	1.62	**13.26**
ribonucleoprotein complex	37	104	0.52	**12.01**
chromosome	27	97	−1.62	**10.08**
spindle	9	47	−0.99	**9.87**
extracellular space	37	57	**-2.23**	0.31
cell junction	38	28	−0.19	−2.17
extracellular region	98	105	**-3.36**	**-3.26**
intrinsic to membrane	307	246	**-2.55**	−7.7

Designated ontologies were selected after the analyses of log-transformed data. Criteria for inclusion were an ontology containing ≥8 genes and having a z-score >2.0 or <-2.0. High and low values for the placebo (Plac) and DHT groups in specific ontologies are highlighted in bold print. Androgen administration also stimulated over 25 different ontologies related to lipid biosynthesis, homeostasis, transport and binding, as previously shown [2].

**Table 8:** DHT impact on KEGG pathways in human meibomian gland epithelial cells.

**KEGG Pathway**	**DHT Genes ↑**	**Plac Genes ↑**	**DHT z-score**	**Plac z-score**
Lysosome	26	7	**5.76**	−1.06
Oxidative phosphorylation	21	8	**4.3**	−0.57
Peroxisome	16	3	**4.24**	−1.48
Aldosterone-regulated sodium reabsorption	9	2	**3.41**	−0.83
Insulin signaling pathway	21	9	**3.35**	−0.82
mTOR signaling pathway	10	3	**3.18**	−0.67
PPAR signaling pathway	12	3	**3.01**	−1.24
DNA replication	1	19	−1.05	**9.94**
Spliceosome	4	39	−1.93	**9.2**
Cell cycle	5	36	−1.53	**8.41**
Mismatch repair	1	11	−0.51	**7.22**
RNA transport	12	27	0.14	**4.27**
p53 signaling pathway	7	15	0.79	**4.03**
Small cell lung cancer	8	17	0.61	**3.89**

Pathways were selected after the analysis of log-transformed data. The criterion for inclusion was a pathway having a z-score >2.0 or <-2.0. High and low values for the placebo (Plac) and DHT groups in specific pathways are highlighted in bold print.

The influence of DHT on immortalized human conjunctival epithelial cells was quite different than that observed in human meibomian gland epithelial cells. As demonstrated in Table 9, DHT enhanced the expression of genes related to epithelium development, regeneration, wound healing, cell migration, Wnt receptor signaling, antioxidant activity and vacuoles, and reduced those associated with translation, RNA processing, mitotic cell cycle, immune response, JAK-STAT cascades, NADH dehydrogenase activity and ribosomes. In addition, androgen administration stimulated KEGG pathways linked to lysosomes, p53 signaling and endocytosis, and suppressed pathways involved with oxidative phosphorylation, the proteosome and RNA transport (Table 10).

**Table 9:** Effect of DHT on the expression of gene ontologies in human conjunctival epithelial cells.

**Ontology**	**DHT Genes ↑**	**Plac Genes ↑**	**DHT z-score**	**Plac z-score**
**Biologic Process**				
apoptosis	141	152	**6.33**	**4.91**
response to stress	215	252	**5.14**	**4.87**
regulation of signal transduction	129	100	**5.02**	−0.55
epithelium development	50	33	**4.95**	0.25
nerve growth factor receptor signaling pathway	33	21	**4.95**	0.74
regeneration	18	9	**4.87**	0.51
wound healing	61	47	**3.95**	0.1
cell proliferation	106	144	**3.17**	**5.15**
cytokine production	32	30	**2.99**	1.36
cell migration	53	41	**2.61**	−0.81
cell adhesion	69	58	**2.46**	−0.79
MAPKKK cascade	35	19	**2.59**	−1.84
canonical Wnt receptor signaling pathway	15	14	**2.32**	1.18
toll-like receptor 4 signaling pathway	10	10	**2.06**	1.39
translation	27	109	−0.12	**13.76**
viral reproduction	19	103	−1.64	**12.88**
innate immune response	33	68	1.36	**6.69**
RNA processing	26	93	−2.41	**6.63**
cell cycle checkpoint	16	43	0.43	**6.36**
macromolecule metabolic process	411	583	0.7	**5.9**
gene expression	189	331	−2.00	**5.18**
immune response	69	103	1.66	**4.27**
mitotic cell cycle	54	82	1.65	**4.2**
JAK-STAT cascade	5	14	0.27	**3.85**
antigen processing and presentation	2	12	−0.98	**3.53**
system process	69	64	**-4.59**	**-7.03**
neurologic system process	57	40	**-3.59**	**-7.13**
regulation of transcription	105	117	**-2.41**	**-3.68**
**Molecular Function**				
protein binding	562	709	**6.93**	**9.48**
enzyme inhibitor activity	44	22	**6.2**	−0.08
SH3 domain binding	21	9	**5.25**	0.05
cell adhesion molecule binding	9	4	**3.85**	0.35
antioxidant activity	10	8	**3.57**	1.89
catalytic activity	374	450	**2.59**	**2.73**
translation initiation factor activity	7	11	**2.09**	**3.63**
voltage-gated ion channel activity	3	9	**-2.8**	−1.65
structural constituent of ribosome	5	69	−1.71	**16.86**
RNA binding	32	153	**-2.89**	**12.32**
threonine-type endopeptidase activity	0	14	−1.22	**9.88**
NADH dehydrogenase activity	0	16	−1.56	**8.37**
small GTPase regulator activity	22	8	0.82	**-3.22**
zinc ion binding	95	98	**-2.84**	**-4.69**
DNA binding	103	137	**-3.53**	**-2.83**
G-protein coupled receptor activity	8	13	**-6.46**	**-6.7**
**Cellular Component**				
vacuole	43	32	**5.27**	1.48
cytoplasm	642	854	**8.33**	**13.06**
intracellular	792	1059	**5.01**	**11.05**
cell surface	43	30	**3.99**	0.03
lysosome	34	32	**4.2**	**2.45**
intracellular organelle part	389	592	**2.42**	**9.7**
organelle	631	924	**2.15**	**11.22**
intrinsic to membrane	293	281	**-2.3**	**-7.61**
ribonucleoprotein complex	18	128	**-2.64**	**14.84**
ribosome	6	68	−1.74	**14.78**
mitochondrion	80	224	−0.25	**13.3**
membrane part	361	366	−1.26	**-6.2**

Specific ontologies were selected after the analyses of log-transformed data. Criteria for inclusion in the Table were an ontology containing ≥8 genes and having a z-score >2.0 or <-2.0. High and low values for the placebo (Plac) and DHT groups in designated ontologies are highlighted in bold print.

**Table 10:** DHT influence on KEGG pathways in human conjunctival epithelial cells.

**KEGG Pathway**	**DHT Genes ↑**	**Plac Genes ↑**	**DHT z-score**	**Plac z-score**
Bacterial invasion of epithelial cells	16	5	**5.02**	−0.81
Lysosome	20	16	**3.89**	1.15
p53 signaling pathway	13	8	**3.69**	0.43
Axon guidance	18	3	**2.89**	−2.97
Endocytosis	25	17	**2.78**	−0.8
Ribosome	1	48	−2.25	**13.98**
Oxidative phosphorylation	7	38	−0.53	**8.25**
Proteasome	2	20	−0.73	**7.76**
Antigen processing and presentation	2	14	−1.35	**3.04**
RNA transport	8	24	−0.98	**2.42**

Pathways were chosen after the analysis of log-transformed data. The criterion for inclusion was a z-score >2.0 or <-2.0. High and low values for the placebo (Plac) and DHT groups in designated pathways are highlighted in bold print.

Of interest, some ontologies were increased in both immortalized cell populations, regardless of treatment, such as cell death and apoptosis. In addition, there were ontologies that were decreased by androgens in both immortalized cells, including cellular component biogenesis, cellular location, innate immune response and nucleic acid metabolic processes. However, the majority of changes in gene ontologies and KEGG pathways appeared to be cell-specific.

## Discussion

The present study demonstrates that androgen treatment significantly influences the expression of thousands of genes in immortalized human meibomian gland and conjunctival epithelial cells. The nature of this DHT action is predominantly cell-specific: some androgen responses are shared by both cell types, the majority are unique, and others are completely opposite. Depending upon the cell type, DHT exerts a significant effect on many gene ontologies and KEGG pathways, including those related to lipid dynamics, innate immunity, cell cycle, JAK-stat cascades, oxidative phosphorylation, the proteasome, and mTOR, Wnt and PPAR signaling. Our findings support our hypothesis that androgens regulate gene expression in human meibomian gland and conjunctival epithelial cells.

Our finding that the nature of DHT action on ocular surface and adnexal cells is predominantly cell-specific is not surprising. It is well established that androgen effects are not necessarily the same in different tissues. For example, androgens increase immunoglobulin A (IgA) and secretory component (SC) expression in the lacrimal gland, appear to have no influence on IgA or SC levels in salivary, respiratory, intestinal, uterine or bladder tissues, and actually decrease IgA amounts in the mammary gland [22,23]. In addition, we have found that testosterone induces a 7.8- to 13-fold increase in epidermal growth factor and nerve growth factor mRNA levels in the submandibular gland [24] but has no effect on these factors in the lacrimal gland (unpublished). Conversely, testosterone stimulates the expression of submandibular androgen-repressed protein (SMARP) in the lacrimal gland, but suppresses SMARP levels in the submandibular gland [25]. As another example, androgens promote the angiogenic activity of prostate epithelial cells, but reduce such activity by prostate stromal cells [26]. In effect, the nature of androgen influence is generally cell- and tissue-specific.

Androgen exposure caused a striking impact on gene expression in immortalized human meibomian gland epithelial cells. Most notable were the effects of DHT on lipid- and keratin-related genes. Androgen treatment induced a significant increase in the activity of numerous genes associated with lipogenesis and cholesterogenesis [2]. This hormone response is analogous to the androgen influence on meibomian glands in vivo [27-30], wherein testosterone stimulates many genes linked to lipid metabolic pathways. Androgen administration also led to a 40 fold decrease in the mRNA level of small proline-rich protein 2A (SPPR2A). This gene, which is significantly upregulated in human meibomian gland dysfunction (MGD) [31], encodes a protein that promotes keratinization [32]; keratinization, in turn, is believed to be a primary cause of MGD and the consequent tear film hyperosmolarity and evaporative dry eye [3]. The SPPR2A gene is also significantly downregulated by androgens in meibomian glands of male and female mice [27,28]. These combined DHT effects, increasing lipogenesis and suppressing keratinization, may begin to explain how topical androgens enhance the synthesis and secretion of meibomian gland lipids, prolong the tear film breakup time and alleviate evaporative dry eye disease [32,33]. In addition, these DHT effects may account for why androgen insufficiency (e.g., during anti-androgen treatment, complete androgen insensitivity syndrome and/or aging) is associated with keratinization of the meibomian gland ductal epithelium (i.e., orifice metaplasia), altered meibum lipid profiles, and a reduced quality of meibomian gland secretions [34-38].

Androgen treatment also led to a significant change in the expression of many other genes in immortalized human meibomian gland epithelial cells, such as those associated with steroidogenesis, microbial protection, tissue development, oxidative stress, mTOR and PPAR signaling, cell cycle, innate immunity and angiogenesis. Androgen administration upregulated the mRNA levels of defensin β1, an antimicrobial peptide implicated in epithelial surface resistance to microbial colonization [39], as well as steroid-5α-reductase, α polypeptide 1, which catalyzes the conversion of testosterone into the more potent androgen, DHT [39]. This steroid regulation appears to be a form of feed-forward control exerted by DHT on its own biosynthesis [40]. Androgen increased the gene expression of leptin receptor, involved in the regulation of fat metabolism, glucose homeostasis, wound healing and the immune system [39]; FOXO1, a transcription factor that mediates cell responses to oxidative stress [39] and is known to interact with androgen receptors [41]; and stearoyl-CoA desaturase, an iron-containing enzyme that catalyzes the synthesis of unsaturated fatty acids. Testosterone enhances stearoyl-CoA desaturase mRNA levels in mouse male and female meibomian glands [27,28], and the targeted disruption of this rate-limiting enzyme causes meibomian gland atrophy [42]. Androgen exposure also increased ontologies and pathways related to peroxisomes, which are organelles involved in metabolism of fatty acids and other metabolites [39]; PPAR, which may promote tissue differentiation [43,44]; and mTOR, a serine/threonine protein kinase that may modulate cell growth, cell proliferation, cell motility, cell survival, protein synthesis and transcription [39,45,46], and is also activated by androgens in the prostate [47]. Androgen administration downregulated genes related to cell cycle regulation (e.g., ubiquitin-conjugating enzyme E2C, cyclin-dependent kinase 1 and cyclin B2), innate immunity (e.g., chemokine (C-X-C motif) ligand 5 and thrombospondin 1) [39,48] and angiogenesis (e.g., thrombospondin 1). Thrombospondin 1 mRNA content is also decreased by androgens in the prostate, bladder and breast cancer cells [49-52]. Also notable was the DHT suppression of gene expression for matrix metallopeptidase 9, an enzyme that is increased in the tear film in dry eye and is known to promote corneal inflammation [53].

The effect of DHT on immortalized human conjunctival epithelial cells was quite different than that observed in human meibomian gland epithelial cells. For example, androgen administration enhanced the expression of genes involved in epithelium development, regeneration, wound healing and cell migration (e.g., matrix metallopeptidase, kallikrein-related peptidases 5, 6 & 11, cystatin E/M, laminin, α3), and suppressed those related to the immune response (e.g., chemokine (C-X-C motif) ligand 6, serpin peptidase inhibitor, clade B, member 4, complement component 1, r subcomponent, interferon-induced protein 44-like, interferon induced transmembrane protein, complement factor B) and mitotic cell cycle (e.g., septin 4, endothelin 1, F-box protein 6 and proteasome subunit, β type, 9). The decrease in immune-related gene activity may play a role in the reported androgen ability to alter the development of allergic conjunctivitis [5] and to attenuate the immune effect of lipopolysaccharide in both conjunctival and meibomian gland epithelial cells [54]. The downregulation of conjunctival genes associated with the cell cycle, which was also found in immortalized human meibomian gland epithelial cells, may reflect a hormone-induced bias toward cell differentiation as compared to proliferation. Androgens are also known to inhibit the cell cycle in other tissues [55-57].

Of particular interest was the DHT upregulation of mucin 16 (MUC16), and downregulation of mucin 1 (MUC1), gene expression in the conjunctival epithelial cells. These transmembrane mucins help to prevent pathogen penetrance into the eye and to maintain a wet ocular surface phenotype [18]. The mucin gene intensities in our study were relatively low, especially for MUC16. This finding may reflect the fact that we cultured cells in serum-free media: exposure of conjunctival epithelial cells to serum, which leads to their stratification, has been reported to promote mucin expression [18]. It is possible that the lack of serum may also have influenced the nature of the MUC1 response to DHT. Thus, others have shown that androgen increases MUC1 expression when breast and prostate cell lines are cultured in serum [58]. This observation would be consistent with the decreased MUC1 levels found in the conjunctiva an individual with complete androgen insensitivity syndrome [12]. We are currently investigating whether the presence or absence of serum causes significant variations in the molecular biologic response of ocular surface and adnexal cells to androgen administration.

Ultimately, it is very important to demonstrate that cellular responses in vitro duplicate those in vivo. Such demonstrations, as we have recently done with androgens and the meibomian gland [2,27-31,34-38], may provide new and meaningful insight into the regulation of ocular surface cells in health and disease.
